# Payment options: do they affect outcome in the critically ill

**DOI:** 10.1186/cc14641

**Published:** 2015-03-16

**Authors:** A Kar, A Datta

**Affiliations:** 1 Medica Superspecialty Hospital, Kolkata, India

## Introduction

Increasing cost is an important issue in critical care medicine. We tried to analyze in a level 3 care ICU in Kolkata of a tertiary care hospital whether the different payment options (self-paying vs. insurance/corporate paying) do affect the outcome in the critically ill.

## Methods

Our prospective study included 1,520 patients admitted consecutively to a level 3 care ICU for a period of 20 months. Readmitted patients during the same period were excluded. Payment method was documented for all and divided into two groups as self-paying and insurance/corporate paying. Outcome assessment was done using the APACHE IV model for all cases. Demographic data, number of observed deaths, predicted mortality rate (PMR), standardized mortality ratio (SMR), average length of stay (ALOS), predicted length of stay, and number of discharge against medical advice (DAMA) were documented for each group. Statistical analysis was carried out using unpaired Student *t *test and *P *< 0.05 was considered significant.

## Results

Of 1,520 patients, 995 (65.46%) cases were self-paying while 525 (34.54%) cases were insurance/corporate paying. In the self-paying group, mean age was 59.65 years ± 17.26 SD (median 62), APACHE IV score mean was 62.50 ± 33.61 SD (median 57), average LOS 4.67 days ± 4.29 SD (median 3), PMR was 22.71, 226 observed deaths, 85 cases of DAMA, and SMR was 1.00 (CI = 0.87 to 1.14). In the insurance/ corporate-paying group, mean age was 61.75 years ± 17.19 SD (median 65), APACHE IV score mean was 58.53 ± 32.94 SD (median 54), average LOS was 5.64 days ± 5.61 SD (median 4), PMR was 21.26, 113 observed deaths, six cases of DAMA, and SMR was 1.01 (CI = 0.83 to 1.21). In the two compared groups, predicted mortality and SMR were not statistically significant (*P *= 0.2808); however, ALOS in the insurance/ corporate paying group was significantly higher than the self-paying group (*P = *0.0002), mean age of the insurance/corporate paying group was significantly higher than the self-paying group (*P = *0.02), and incidence of DAMA is significantly higher in the self-paying group (8.54%) as compared with insurance/corporate paying group (1.14%). Root-cause analysis showed DAMA cases are mostly financial (>95%).

## Conclusion

Statistically significant differences in ALOS and DAMA in the two groups are probably due to cost of healthcare not affordable to all.

